# Substantial non‐homologous recombination and structural variation results from *Brassica* AABC and CCAB hybrid meiosis

**DOI:** 10.1111/tpj.70555

**Published:** 2025-10-31

**Authors:** Zhenling Lv, Shima Mahmoudi, Annaliese S. Mason

**Affiliations:** ^1^ Plant Breeding Department, INRES University of Bonn Kirschallee 1 53115 Bonn Germany

**Keywords:** non‐homologous recombination, homologous recombination, crossover frequency, *Brassica napus*, *Brassica carinata*, *Brassica juncea*, interspecific hybrids, reduced gametes, unreduced gametes, introgression breeding

## Abstract

Meiotic crossovers contribute to genetic diversity and play a crucial role in homologous chromosome segregation. Non‐homologous crossovers in *Brassica*, involving the exchange of genetic material between genomes, can be valuable for transferring novel traits or characteristics between *Brassica* species. However, there are a limited number of studies that specifically investigate crossover frequencies in populations of interspecific hybrids. We investigated the distribution and frequency of homologous crossover events, as well as non‐homologous recombination and structural variation, in hybrids between *B. juncea* (AABB) × *B. napus* (AACC) (resulting in AABC hybrids; 5 genotypes) and *B. napus* (AACC) × *B. carinata* (BBCC) (resulting in CCAB hybrids; 4 genotypes). The analysis was performed on individuals derived from microspore culture of both unreduced and reduced gametes produced by the AABC and CCAB hybrids. All AABC and almost all CCAB unreduced gamete‐derived individuals and most AABC and CCAB reduced gamete‐derived individuals showed copy number variation indicative of non‐homologous (A–C) recombination. Additionally, a higher frequency of homologous crossovers, also in centromeric and pericentromic regions, was observed in the diploid genomes of the AABC and CCAB hybrids. Overall, these hybrid types show high frequencies of A–C introgressions, which may be useful in *B. juncea* or *B. carinata* introgression breeding, and this increased recombination frequency may help break up existing linkage disequilibrium blocks in the *Brassica* A and C genomes.

## Significance Statement

We show that hybrids between *Brassica* crop species with different combinations of genomes and chromosomes undergo substantial shuffling of these genomes and chromosomes following meiosis, which may help accelerate breeding progress.

## INTRODUCTION

The fundamental process of meiotic recombination via crossovers (COs) is pivotal for generating genetic diversity in all sexually reproducing organisms (Blary et al., 2018; Mercier et al., 2015; Mézard et al., 2015). Crossovers between homologous chromosomes play a crucial role in ensuring accurate segregation of these chromosomes into daughter gametes, and allow alleles belonging to both chromosomes to be recombined (shuffled) in the progeny (Choi & Henderson, 2015). The presence of at least one obligate crossover per homologous chromosome pair is essential for accurate homolog segregation and genomic stability, and hence crossover frequency and distribution are strictly regulated: typically, only one to three crossovers form per homologous chromosome pair (reviewed by Mercier et al., 2015). Crossovers are also not uniformly distributed across chromosomes; generally, crossover frequencies increase toward the telomeres, while the centromeric and pericentromeric regions lack crossovers entirely in most species, resulting in a U‐shaped distribution of crossover frequency along the chromosome axis (Mercier et al., 2015; Mézard et al., 2015).

The recombination of alleles via homologous recombination is critical for plant breeding, which aims to optimize the specific combination of alleles present within a single cultivar (Choi & Henderson, 2015; Mercier et al., 2015). As well as crossovers between homologous chromosomes, crossovers between non‐homologous chromosomes are often also of interest. Specifically, crossovers between non‐homologous chromosomes are required for introgression breeding, that is, the transfer of useful traits from wild relative species into crops, which generally occurs via the transfer of a recombined chromosomal segment. Interspecific hybridization can greatly improve genetic diversity within crop germplasm and can introduce novel traits into breeding programs (reviewed by Katche et al., 2019, Adonina et al., 2021). Hence, progress in plant breeding is often limited by the constraints imposed by chromosomal crossovers, both homologous and non‐homologous.

*Brassica napus* (AACC, 2n = 38) is an economically important oilseed crop, contributing approximately 13–16% of the world's vegetable oil (Hu et al., 2022; Wang et al., 2018). The origins of *B. napus* can be traced back to the Mediterranean region around 7500 years ago, where it emerged through natural hybridization between two diploid progenitors, *B. rapa* (AA, 2n = 20) and *B. oleracea* (CC, 2n = 18) (Chalhoub et al., 2014; Liu et al., 2014; Lysak et al., 2005; Wang et al., 2011; Yu et al., 2017). As a result of its status as a recent allopolyploid species, possessing only a fraction of the genetic diversity present in the diploid progenitors and present only as a domesticated crop, and also as a result of intensive breeding selection for oil‐quality traits, rapeseed is considered highly inbred (Chalhoub et al., 2014; Cowling, 2007; Mason & Snowdon, 2016). Hybridization events between *Brassica napus* and progenitor species *B. rapa* and *B. oleracea* are thought to have played a major role in the domestication and evolution of rapeseed (Wang et al., 2023), and hybridization between different *Brassica* species has frequently been carried out for crop improvement (reviewed by Katche et al., 2019). However, the majority of genetic diversity and useful traits existing in *Brassica* species and wild relatives have yet to be exploited for crop improvement (reviewed by Quezada‐Martinez et al., 2021).

Boosting frequencies of homologous and non‐homologous recombination is of clear interest for crop improvement, particularly in inbred species such as *B. napus*. Limited numbers of crossovers per chromosome and prevention of crossovers in centromeric and pericentromeric regions are conserved features of recombination across most crop species, including *Brassica rapa* (Pelé et al., 2017), *Brassica oleracea* (Cai et al., 2023) and *Brassica napus* (Boideau et al., 2021), but this limits breeding gains by limiting the possible allelic combinations that can be achieved by meiotic recombination. Homoeologous crossovers (between ancestrally homologous sequences derived from different species or subgenomes) are usually prevented in established allopolyploids, putatively because homoeologous recombination can lead to aneuploidy and loss of genetic information and hence loss of fertility and viability (Gaeta & Chris Pires, 2010; Pelé et al., 2018). Frequent homoeologous recombination is mostly prevented in established allopolyploid *B. napus*, putatively by a combination of quantitative genetic factors (Jenczewski et al., 2003; Liu et al., 2006), likely allelic variants of specific meiosis genes (Higgins et al., 2021). However, homoeologous recombination is very commonly observed in synthetic *Brassica* hybrids of different types (reviewed by Gaebelein & Mason, 2018, Ihien Katche & S. Mason, 2023), where the frequency is also affected by genetic factors (Gaebelein, Schiessl, et al., 2019; Katche, Schierholt, Becker, et al., 2023; Katche, Schierholt, Schiessl, et al., 2023; Quezada‐Martinez et al., 2022). Homoeologous recombination is also very common between the closely related A and C genomes in *Brassica*, but less common between the more distantly related B genome and the A/C genomes (Gaebelein, Alnajar, et al., 2019; Mason et al., 2010). Homoeologous recombination has also been shown to generate novel genetic diversity (Gaeta et al., 2007; Rousseau‐Gueutin et al., 2017; Song et al., 1995; Szadkowski et al., 2010) and to affect agronomically interesting traits such as flowering time (Schranz & Osborn, 2000) and seed quality (Stein et al., 2017) in *Brassica* and in other polyploid species (reviewed by Schiessl et al., 2019).

An interesting phenomenon whereby crossover frequencies are boosted by genome structure has been observed previously in *Brassica*. In allotriploid AAC hybrids (*B. rapa* × *B. napus*), a higher number of crossovers (type I COs, as identified by MLH1 and HEI10 antibody staining) is observed in the diploid AA genome compared with natural *B. napus* (AACC) and the strict regulation of COs can be modified both in frequency and distribution, with COs occurring in typically cold regions at distances as close as 375 kb from the centromere in allotriploids (Boideau et al., 2024; Leflon et al., 2010). In AA hybrids containing different additional C‐genome chromosomes (zero, one, three, six or nine additional C chromosomes), specific unpaired C genome chromosomes also significantly affected recombination frequency and reduced crossover interference (Suay et al., 2014). As well, the presence of chromosome C9 of *B. oleracea* in these hybrids was found to promote additional crossovers near the pericentromeric regions, while C9 in *B. napus* had no significant effect, despite possessing a similar set of meiotic genes (Pelé et al., 2025). Recently, similar alterations in crossover frequency and distribution were observed in the diploid genomes of pentaploid wheat hybrids (Yang et al., 2022), putatively also due to the presence of an additional haploid genome. These results suggest that this crossover boost phenomenon may have wider generalizability, even outside *Brassica* species, although the exact mechanisms underlying this effect are still unknown.

Unreduced gametes refer to gametes that retain the same chromosome number as the somatic cells, rather than undergoing a normal reduction division during meiosis. Unreduced gametes appear to be produced at higher frequencies in interspecific hybridization events and by interspecific hybrids (Kreiner et al., 2017; Ramsey & Schemske, 1998), and may be selected for by microspore culture (Mason, Nelson, Castello, et al., 2011; Nelson et al., 2009) and elevated in frequency by temperature changes in *Brassica* (Mason, Nelson, Yan, & Cowling, 2011). Putatively, the production of unreduced gametes may be a mechanism which has been co‐opted (exapted) to facilitate polyploid formation (Mason & Pires, 2015). There is also some evidence that unreduced gametes may show different recombination structures to reduced gametes in *Brassica* (Szadkowski et al., 2011). However, this phenomenon, and its genetic effects, is relatively understudied in most taxa (Mason & Pires, 2015), including *Brassica*.

In this study, we aimed to investigate homologous and non‐homologous crossover frequency in *B. juncea* × *B. napus* AABC hybrids (five genotypes) and *B. napus* × *B. carinata* CCAB hybrids (four genotypes), derived from both unreduced and reduced gametes produced by the F_1_ hybrids (see Mason et al., 2014 for details of the experimental material). We found almost all AABC and CCAB hybrids showed extensive chromosome variation, predominantly resulting from non‐homologous (A–C) chromosome interactions in Meiosis I. Additionally, a higher frequency of homologous crossovers, also in centromeric and pericentromic regions, was observed in the diploid genomes of the AABC and CCAB hybrids. This high frequency of A–C introgressions could be particularly advantageous for introgression breeding in *Brassica juncea* (AABB) and *Brassica carinata* (BBCC) and increased recombination frequency may also help break up existing linkage disequilibrium blocks in the *Brassica* A and C genomes.

## RESULTS

### Substantial chromosomal structural variation was observed in unreduced gamete‐derived AABC‐ and CCAB hybrids

**Figure 1:**
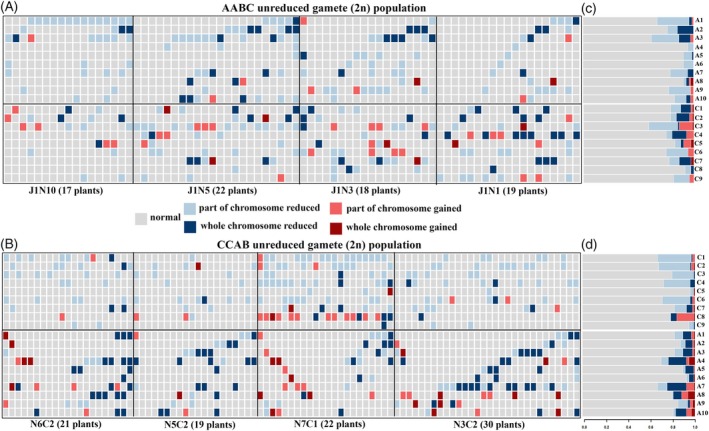
Substantial chromosome structural variation in unreduced gamete‐derived AABC and CCAB hybrid populations. (A) Each row represents an individual chromosome (A1–A10, C1–C9) from the AABC_unreduced gamete‐derived population, and each column represents a distinct individual. (B) Each row represents an individual chromosome (C1–C9, A1–A10) from the CCAB_unreduced gamete‐derived population, and each column represents a distinct individual. (C) Summary of the data shown in panel A, illustrating the relative proportions of different chromosome types within the AABC_unreduced gamete‐derived population. (D) Summary of the data shown in panel B, illustrating the relative proportions of different chromosome types within the CCAB_unreduced gamete‐derived population.

Despite the putative inheritance of a complete AABC or CCAB chromosome set as a result of unreduced gamete formation, we detected a very high frequency of novel variation in chromosome number and structure in the AABC and CCAB unreduced gamete‐derived hybrids (Figure 1). Based on our analyses, regions with copy numbers above a specific threshold were marked as duplications, while those below the threshold were marked as deletions, and all other regions were considered to have normal copy number (Figure S3). In the AABC unreduced gamete‐derived population, all individuals (76/76 across four different genotype combinations) showed loss or gain of at least one whole chromosome or chromosome segment (Figure 1A and Table S5). No significant difference was observed in the frequency of copy number variation between the haploid C subgenome and the diploid A subgenome in the AABC unreduced gamete‐derived population (*χ* ^2^‐test, *P*‐value = 0.17) (Figure 1C and Table S5), and for both A and C subgenomes in the AABC unreduced gamete‐derived population, chromosome deletion events were significantly more common than chromosome duplication events (*χ* ^2^‐test, *P*‐value <2.2e‐16) (Figure 1C and Table S5). Additionally, different chromosomes within different subgenomes displayed varying degrees of copy number variation (*χ* ^2^‐test, *P*‐value = 3.064e‐06). In the A subgenome, chromosomes A1, A2, and A3 exhibited a greater frequency of chromosome deletion events, while in the C subgenome, chromosomes C3, C6, and C7 showed higher frequencies of chromosomal deletion (Figure 1C and Table S5). Chromosome duplication events primarily occurred within the C subgenome in the AABC unreduced gamete‐derived population (*χ* ^2^‐test, *P*‐value = 2.713e‐05), with a notable concentration on C3 and C5 (Figure 1C and Table S5).

Similarly, nearly all individuals in the CCAB unreduced gamete‐derived population (89 out of 92 across four genotype combinations) exhibited at least one chromosome or chromosome segment copy number variation event (Figure 1B and Table S6). In this population, there is no significant copy number variation frequency difference in the A subgenome compared with the C subgenome (*χ* ^2^‐test, *P*‐value = 0.191) (Figure 1D and Table S6). For both the A and C subgenomes in the CCAB unreduced gamete‐derived population, chromosome deletions were significantly more frequent than duplications (*χ* ^2^‐test, *P*‐value <2.2e‐16) (Figure 1D and Table S6). Additionally, as observed in the AABC unreduced gamete‐derived population, chromosomes from different subgenomes displayed varying degrees of chromosome variation (*χ* ^2^‐test, *P*‐value = 1.666e‐10). In the A subgenome, chromosomes A4 and A7 had a higher frequency of deletions, while in the C subgenome, chromosomes C1, C2, and C6 showed an increased frequency of deletions (Figure 1D and Table S6). Chromosome duplications were highest on chromosomes A8, A10, A7 and A4 and C8 within the CCAB unreduced gamete‐derived population (Figure 1D and Table S6).

### Chromosome variation events in the diploid genomes of AABC and CCAB reduced gamete‐derived populations

**Figure 2:**
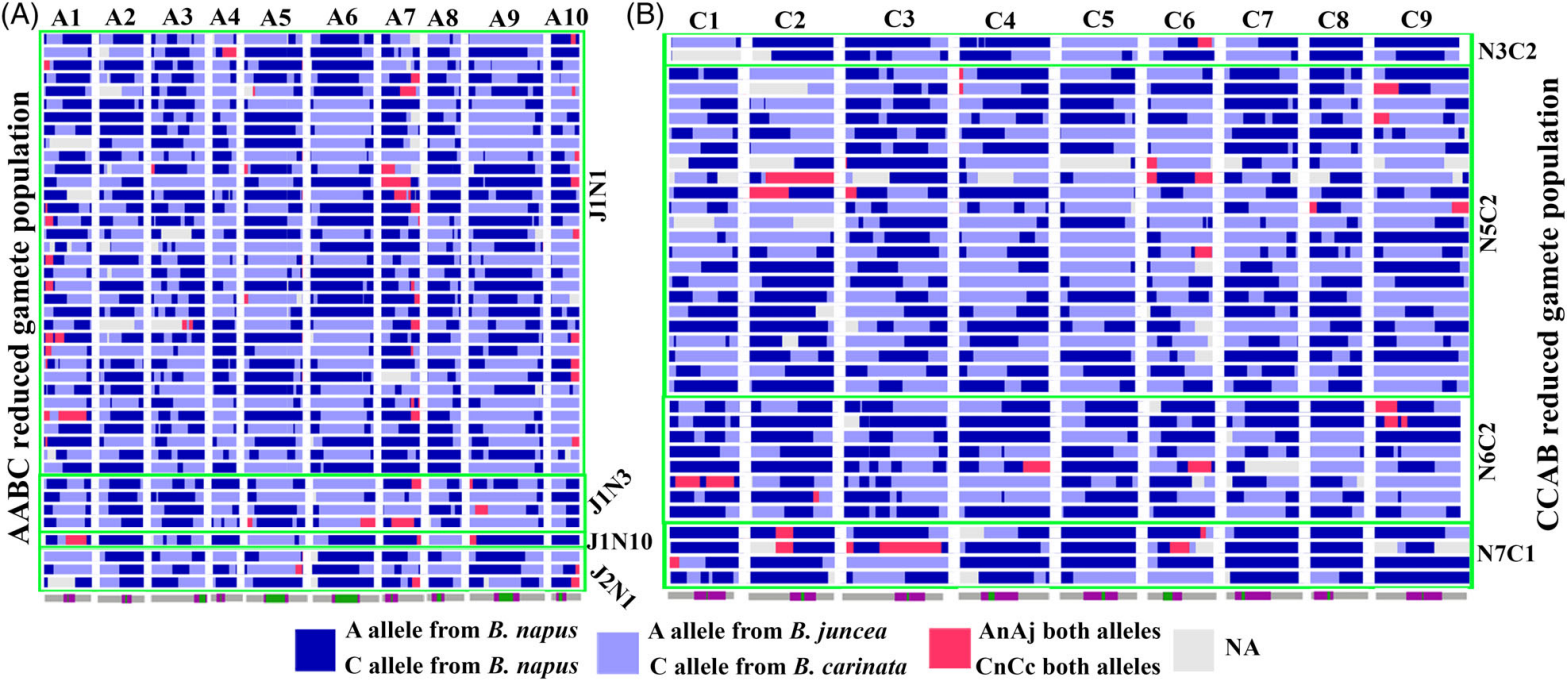
Haplotype‐based allele inheritance profiles in the (A) AABC and (B) CCAB reduced gamete‐derived populations. A^n^ alleles from *B. napus* and A^j^ alleles from *B. juncea* in A^j^A^n^B^j^C^n^‐derived hybrids are represented in dark and light blue, respectively, and C^n^ alleles from *B. napus* and C^c^ alleles from *B. carinata* in C^n^C^c^A^n^B^c^‐derived hybrids also in dark blue and light blue, respectively. The pink regions indicate presence of alleles from both parents (A^n^ + A^j^ or C^n^ + C^c^), while gray regions “NA” represent absence of both parental alleles: these two types of events putatively result from non‐homologous chromosome recombination. The bottom track represents the length of each chromosome, with green indicating the centromere regions and purple representing the pericentromeric regions.

**Figure 3:**
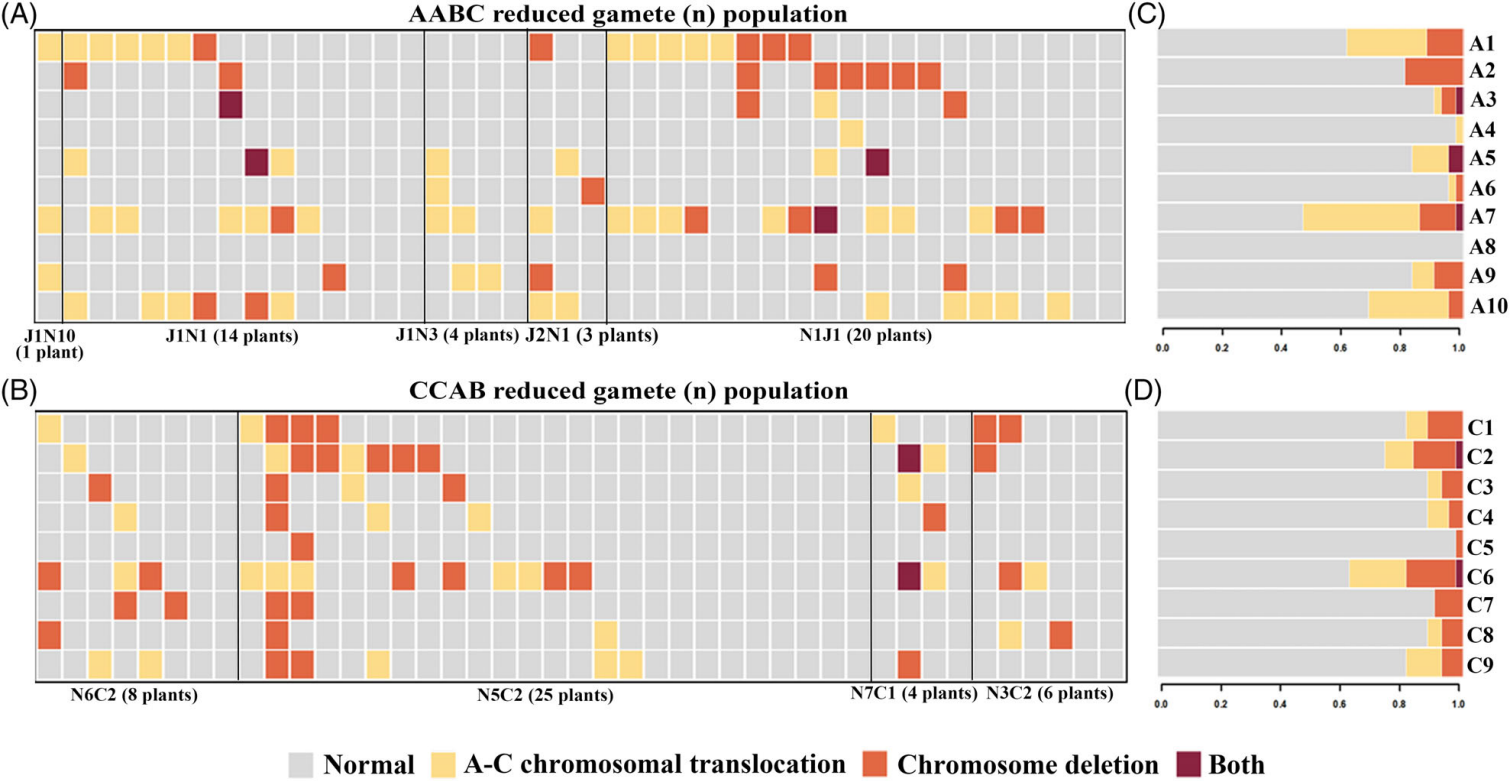
Chromosome translocation and deletion events in the AABC and CCAB reduced gamete‐derived populations. (A) Chromosome‐by‐individual distribution of events in the AABC reduced gamete‐derived population and (B) chromosome‐by‐individual distribution of events in the CCAB reduced gamete‐derived population, where each row represents an individual chromosome (A1–A10, C1–C9), and each column represents a distinct individual. (C) Summary of the frequency of translocations and deletions observed per A genome chromosome in the AABC reduced gamete‐derived population. (D) Summary of the frequency of translocations and deletions observed per C‐genome chromosome in the CCAB reduced gamete‐derived population.

In the AABC and CCAB reduced gamete‐derived population, heterozygous or missing regions of chromosomes from the diploid genomes indicate non‐homologous recombination events and putative translocations between subgenomes (Mason et al., 2015). Interestingly, after imputation, haplotype‐based recombination profiles revealed a proportion of unexpected F_1_‐like patterns. In theory, chromosomes in reduced AABC and CCAB gametes should originate from either the maternal A^j^ or paternal A^n^ allele in the A^j^A^n^BC hybrid (or maternal C^n^ or paternal C^c^ allele in the C^n^C^c^AB hybrid), but not both. The presence of both alleles—or neither (resulting in NA values)—may indicate chromosomal anomalies, such as recombination, translocations, or deletions. To our surprise, such heterozygous and missing regions were present in the majority of individuals in the AABC and CCAB reduced gamete‐derived populations (Figure 2A,B). Among the chromosome translocations we observed, many occurred at the chromosome ends, typically involving a single chromosome break. However, we also found numerous translocations occurring in the middle of the chromosome, which implies that two chromosome breaks are required for the translocation to take place (Figure 2A,B). In the AABC reduced gamete‐derived population, 36/42 individuals (85.7%) showed at least one chromosomal translocation or deletion event, with the most frequent chromosome rearrangements involving chromosome A7 and A1 (Figure 3A,C and Table S7). Similar trends were observed in the CCAB reduced gamete‐derived population, with 29/43 (67.4%) individuals showing evidence of chromosome arrangements, most frequently involving chromosome C6 and C2 (Figure 3B,D and Table S8). Haplotype‐based allele inheritance profiles are also shown in unreduced gamete‐derived populations (see Figure S4).

### Genome‐wide elevation in recombination events and breakpoint distributions

**Figure 4:**
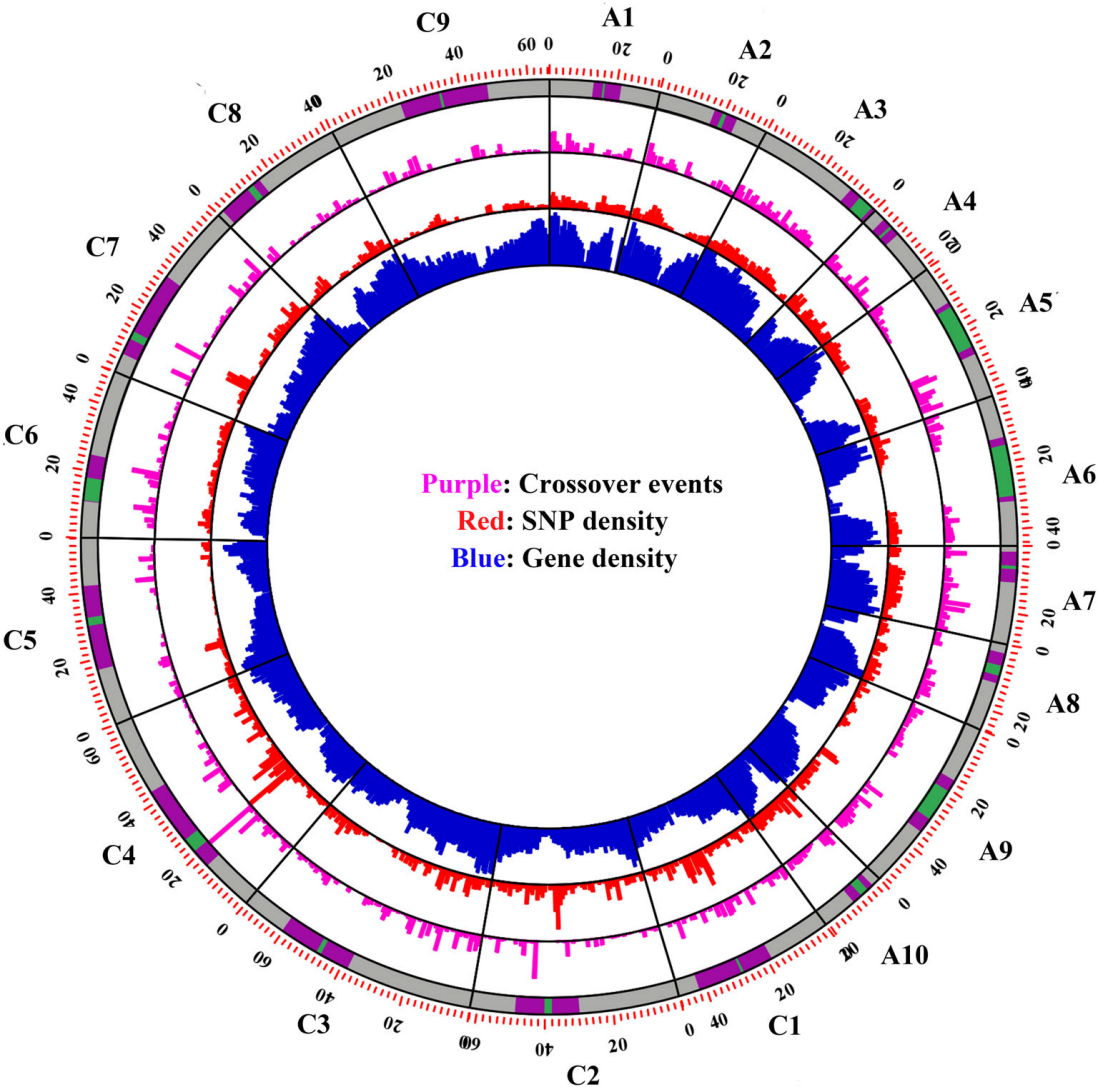
Circos plot of spatial distribution of crossover (CO) events along the AA or CC genomes in *Brassica* AABC and CCAB hybrid‐derived populations. From the innermost to the outermost rings: the blue represents gene density, the red shows SNP density, and the purple indicates the total number of crossover (CO) events detected within 1 Mb windows. The outermost layer depicts chromosomal architecture, with green marking the centromeric regions, purple indicating the pericentromeric regions, and gray representing the overall chromosomal framework (data from reference genome Darmor v. 10; Boideau et al., 2022).

The mean number of putative homologous breakpoints in the A genome (after excluding putative non‐homologous breakpoints, see methods) was 23.7 in reduced (n) and 22.0 in unreduced (2n) lines of the AABC gamete‐derived populations, respectively. In contrast, the C genome of the CCAB population exhibited 17.2 breakpoints in reduced (n) and 21.9 in unreduced (2n) gamete‐derived populations, respectively (Figure S5; Tables S9 and S10). To examine the spatial distribution of meiotic crossovers (COs), we combined CO positions from reduced (n) and unreduced (2n) datasets and calculated raw counts in fixed 1 Mb windows without any additional smoothing or normalization (Figure 4 and Figure S6). In both the AA and CC genomes, crossover events were predominantly enriched along the chromosomal arms, consistent with previously reported patterns in *Brassica* species. However, notable deviations were observed in the CC genome. Specifically, chromosomes C1, C2, C4, C6, C7, C8, and C9 exhibited elevated CO frequencies within the centromeric and pericentromeric regions.

**Figure 5:**
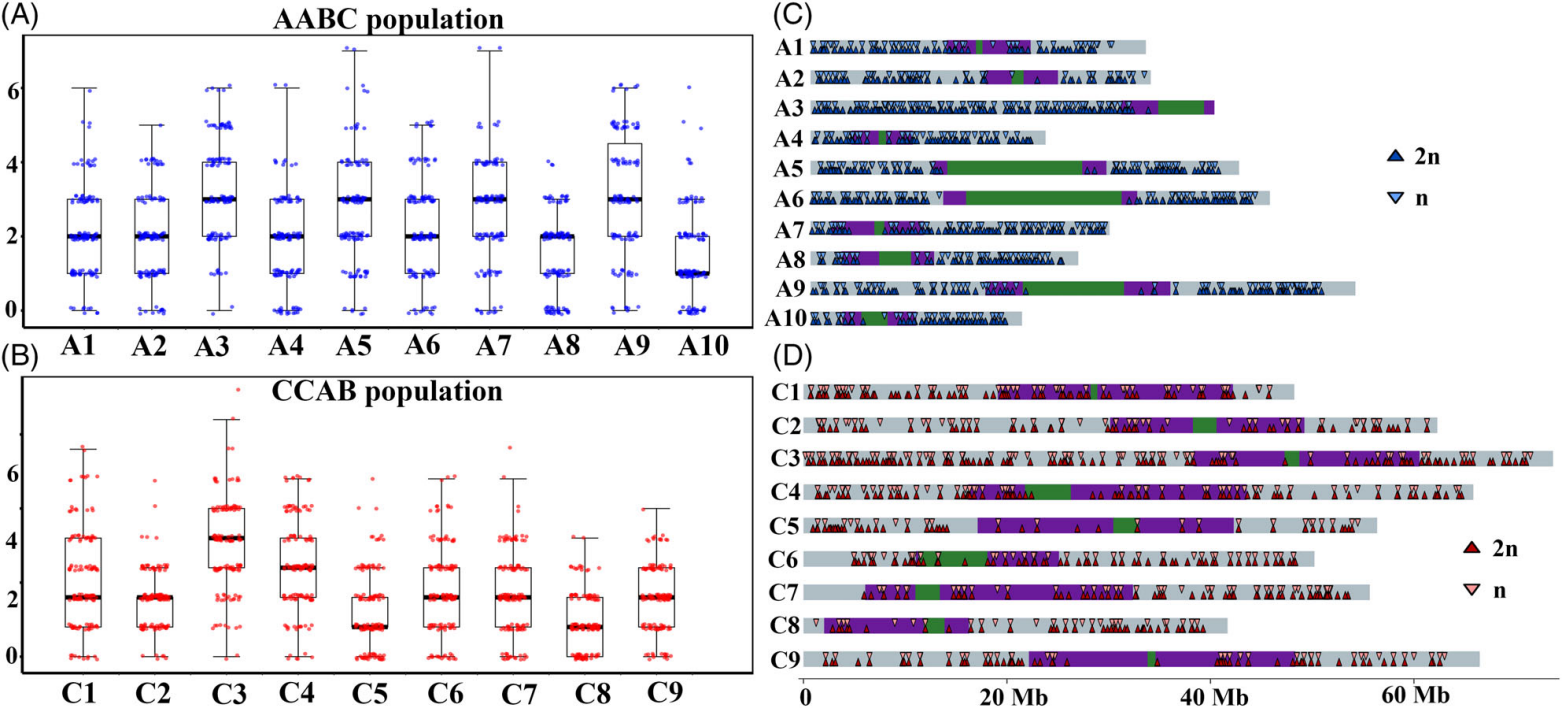
Genome‐wide crossover frequencies in *Brassica* AABC and CCAB hybrid‐derived populations. (A) chromosome‐specific crossover frequencies in the A genome of the AABC population, and the black line in the middle of the box represents the average value. (B) chromosome‐specific crossover frequencies in the C genome of the CCAB population, and the black line in the middle of the box represents the average value. (C) Genome‐wide homologous crossover distribution along the AA genome chromosomes in the AABC population. (D) Genome‐wide homologous crossover distribution along the CC genome chromosomes in the CCAB population. The green region represents the centromere, the purple region indicates the pericentromere, and the gray region corresponds to the chromosomal framework (derived from the Darmor v. 10 reference genome; Boideau et al., 2022). Dark blue and light blue correspond to the reduced (n) and unreduced (2n) populations of AABC, respectively; dark red and light red correspond to the reduced (n) and unreduced (2n) populations of CCAB, respectively.

After determining that the overall crossover number per individual was high, we also wanted to find out how these crossovers were distributed across different chromosomes, and if there was any chromosome specificity or bias. Our analysis indicated that the crossover number increased across all chromosomes, with no apparent chromosome specificity (Figure 5A,B). Up to six or seven crossover breakpoints were observed for some chromosomes (e.g., A5 and A7 in the AABC and C1 and C3 in the CCAB genome) in some individuals (Figure 5A,B). We did not observe a strong positive correlation between chromosome length and crossover frequency, which was unexpected (Figure S7), although crossovers were quantitatively enriched toward the telomeres, as expected. We removed crossover events that occurred at identical positions to generate a dataset of unique crossovers, derived from the previously merged reduced (n) and unreduced (2n) datasets. Based on these unique crossovers, the chromosomal distribution revealed a clear shift in crossover patterns. Crossover events were detected in the centromeric and pericentromeric regions of all AA and CC chromosomes (Figure 5C,D).

### Genotype‐specific effects on global crossover frequency

**Figure 6:**
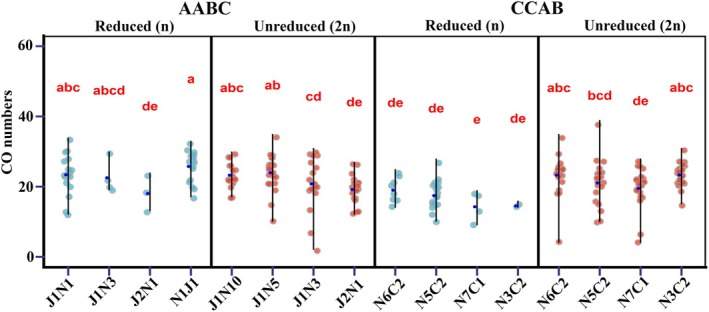
Genotype‐specific effects on the overall frequency of crossovers in *Brassica* AABC and CCAB interspecific hybrids. Light blue indicates populations derived from reduced gametes, and red indicates populations derived from unreduced gametes; each dot indicates one individual. The blue dot in the middle of the black line represents the average value. Statistically significant differences were observed between lines (one‐way ANOVA, *P* = 1.54e‐07); different letters indicate significant differences (Least Significant Differences test, *P* < 0.01).

Despite the low numbers of individuals belonging to each genotype combination, we observed significant differences between progeny sets in the AABC reduced and CCAB reduced and unreduced gamete‐derived populations (Figure 6). In the reduced and unreduced gamete‐derived AABC population, J1N1, J1N5, and J1N10 had higher numbers of crossovers and J2N1 had lower numbers (Figure 6). Of the four different genotype combinations in the CCAB population N7C1 had the lowest crossover number, and N6C2 and N3C2 had significantly higher numbers of crossovers (Figure 6). In genotypes N6C2 and N3C2, unreduced gamete‐derived individuals (*n* = 21 and *n* = 30, respectively) showed higher crossover frequencies than reduced gamete‐derived individuals (*n* = 8 and *n* = 2 respectively, *P* = 0.006 and 0.000172), but for other genotypes the difference between reduced and unreduced gamete‐derived individuals was not significant.

## DISCUSSION

We analyzed a pre‐existing Illumina Infinium 60 K *Brassica* array SNP genotyping dataset for AABC‐ and CCAB hybrid microspore‐derived or test cross individuals (described in Table S1 of Mason et al., 2016) and found 76/76 (100%) of unreduced gamete‐derived AABC and 89/92 (96.7%) of unreduced gamete‐derived CCAB individuals showed at least one partial or whole chromosome with copy number variation in the A/C subgenomes. A similar phenomenon was also observed in the reduced gamete‐derived population, in which 36/42 (85.7%) of AABC and 29/43 (67.4%) of CCAB individuals showed copy number variation putatively resulting from non‐homologous (A‐C) chromosome interactions in meiosis I. Additionally, a high frequency of homologous crossovers, also in centromeric and pericentromic regions, was observed in the diploid genomes of the AABC and CCAB hybrids. Our results suggest that these hybrid types show high frequencies of A–C introgressions, which may be particularly useful in *B. juncea* (AABB) or *B. carinata* (BBCC) introgression breeding, and that this increased recombination frequency in the pericentromeric and centromeric regions may also help break up existing linkage disequilibrium blocks in the *Brassica* A and C genomes.

In our study of the AABC and CCAB gamete‐derived hybrids we observed that almost all individuals exhibited varying degrees of chromosome variation, with the most prominent being the deletion or gain of certain (partial) chromosomes, which was particularly prevalent at the ends of chromosomes. The primary cause of extensive chromosome rearrangements such as loss or gain of chromosome segments is predicted to be homoeologous interactions between the *Brassica* A and C genomes, which are known to be closely related (Parkin et al., 1995, 2003) and to undergo frequent recombination in interspecific hybrids (Leflon et al., 2010; Mason et al., 2010, 2014; Mason, Nelson, Castello, et al., 2011; Nicolas et al., 2009), allohaploids (Boideau et al., 2024; Suay et al., 2014), and even in the established allotetraploid species *B. napus* (Chalhoub et al., 2014; Mason et al., 2016; Osborn et al., 2003). By contrast, the B genome is more rarely involved in non‐homologous recombination with the A or C genomes, although crossovers are known to form between the A/C and B genomes at low frequencies (e.g., 0.5 crossovers per PMC in AABC and CCAB hybrids; Mason et al., 2010; 0.3 crossovers per PMC in ABC hybrids, Gaebelein, Alnajar, et al., 2019). We were unable to assess B/A‐C recombination frequencies in our present study due to limitations of the array data available (SNPs specific to A and C genomes), but we would expect non‐homologous recombination involving the B genome to be much less than that observed between the A and C. The homoeology between the chromosomes in the A and C genomes is well‐characterized (Cheng et al., 2013; Parkin et al., 2003) and the conserved genomic regions or segments that have a similar gene order and content are the most likely to undergo recombination (Mason et al., 2014; Szadkowski et al., 2011; Xiong et al., 2011). This frequent non‐homologous recombination is also known to generate novel genetic diversity and gene combinations as well as new traits (Schranz et al., 2006; reviewed by Schiessl et al., 2019). Hence, in the context of *Brassica* breeding, the utilization of AABC and CCAB hybrids presents a promising avenue for geneticists and plant breeders to augment genetic diversity by creating fresh allelic combinations and to accelerate the introgression of agronomical traits of interest between the allotetraploid *Brassica* species.

Our results suggested an increase in recombination in the pericentromeric and centromeric regions in the AABC and CCAB interspecific hybrids. Similar results were also observed in *Brassica* interspecific hybrids with genome complement AAC (Boideau et al., 2021; Pelé et al., 2017): in these allotriploid AAC hybrids (*B. rapa* × *B. napus*), an increased number of chromosomal crossovers—confirmed by MLH1 or HEI10 antibody staining—was primarily attributed to a reduction or suppression of interference between Class I crossovers. As for Class II crossovers, there is currently no effective antibody available for detecting *Brassica* MUS81, although analysis of the expression levels of Class II‐related genes revealed no significant differences (Boideau et al., 2024). However, in our material, the high frequency of chromosomal structural variation, caused by frequent non‐homologous recombination, may contribute to increased genome instability and disrupt normal gene expression. This genomic instability could potentially comprise another factor leading to an increased number of crossovers, and may even alter their distribution—potentially allowing crossovers to occur in pericentromeric and centromeric regions. We also cannot exclude that some of the crossovers that we observed are due to segregating translocations which were present between the parent genotypes, as has been observed previously in interspecific hybrids between allotetraploid *Brassica* species (Mason et al., 2015). This is usually detectable as breakpoints occurring at exactly the same chromosomal location in multiple individuals: although we did observe such “peaks” (Figure 4 and Figure S6), this phenomenon is unlikely to be responsible for all crossovers observed in the centromeric and pericentromeric regions in our study. Regardless, the putative alterations in crossover distributions and increased crossover frequencies in centromeric and pericentromeric regions in the AABC and CCAB populations observed in our study require further investigation and validation in comparison to genotype‐specific controls. However, these findings could be important for plant breeding strategies and for the development of new varieties with desirable traits. Specifically, by studying crossover frequency genome‐wide in large segregating AABC and CCAB populations, a certain reshaping of crossover patterns and the distribution of crossover events was found, which might help break linkage disequilibrium blocks in centromeric and pericentromeric regions and enhance the generation of new diversity in future plant breeding efforts.

We also observed genotype‐specific differences in crossover frequency between different hybrid types. This finding aligns with previous cytological evidence of non‐homologous recombination frequency differences in AABC hybrids (Mason et al., 2010) as well as marker‐based evidence for non‐homologous recombination frequency in CCAB hybrids (Mason, Nelson, Castello, et al., 2011). Our results suggest that it may be profitable to select and target specific genotypes with higher crossover frequencies for enhanced genetic recombination in breeding programs.

As well as crossovers resulting from novel recombination events between the A and C genomes in the AABC and CCAB hybrids, there are likely other factors contributing to the observed chromosome rearrangements. For example, in the AABC reduced gamete‐derived population, we observed extensive chromosomal variation in the J1N1 genotype, particularly involving chromosome A7. Notably, N1, which corresponds to “Surpass400_024DH”, has already been reported in Mason et al., 2015 to be carrying an A7/C6 reciprocal chromosomal translocation. This leads to overestimation of non‐homologous crossover frequencies due to segregation of this pre‐existing event in the progeny (Mason et al., 2015), but it can also precipitate additional chromosome translocation events (Mwathi et al., 2017), further disrupting genome stability and contributing to elevated chromosomal variation. Interestingly, in two genotype combinations (N6C2 and N3C2), unreduced gametes exhibited higher crossover frequencies than reduced gametes. However, given the limited number of reduced‐gamete plants analyzed (eight and two, respectively), additional data will be required to confirm whether this pattern is consistent across genotypes. Genotype‐specific responses to microspore culture (Takahira et al., 2011) and in unreduced gamete production (Mason, Nelson, Castello, et al., 2011) most likely contributed to our unbalanced experimental design. Additionally, natural allelic variation in meiosis genes, such as *REC8* in barley (Dreissig et al., 2020), could also influence crossover distribution and frequency. Beyond these known factors, there remains much to explore to fully understand the regulatory mechanisms shaping recombination patterns.

## MATERIALS AND METHODS

### Plant material

**Figure 7:**
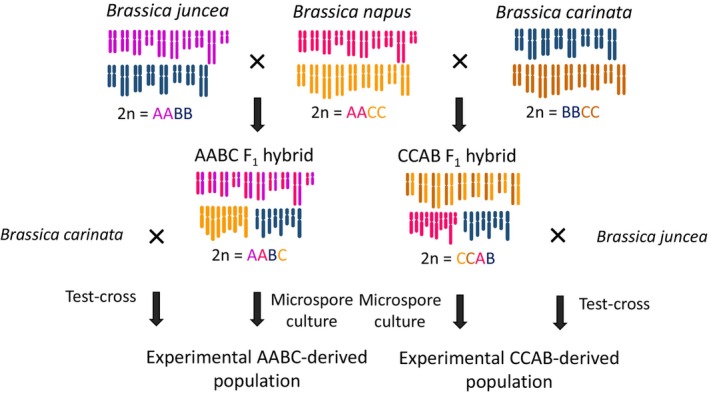
Production and chromosome complements of the parents of the experimental *Brassica* interspecific AABC and CCAB F_1_ hybrid‐derived populations. Gametes isolated by test crossing or microspore culture from five genotypes of F_1_ AABC hybrids (*B. juncea* × *B. napus*) and four genotypes of F_1_ CCAB hybrids (*B. napus* × *B. carinata*) were assessed to determine homologous and non‐homologous crossover frequencies in the F_1_ AABC and F_1_ CCAB meiosis.

**Figure 8:**
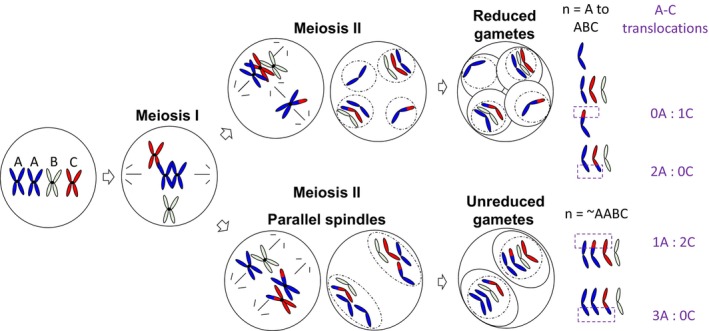
Graphical representation of meiosis with one chromosome per haploid genome, showing unreduced gamete formation via parallel spindles and subsequent chromosomal structural variations and translocation events observed in an example *B. juncea* × *B. napus* AABC hybrid‐derived population.

Interspecific hybrids with the genome complement AABC were previously produced by the cross *B. juncea* × *B. napus*, and interspecific hybrids with the genome complement CCAB were previously produced by the cross *B. napus* × *B. carinata* (Mason, Nelson, Castello, et al., 2011) (Figure 7). Parental *Brassica* genotypes used to produce these hybrids were *B. napus* “Surpass400_024DH,” designated as N1, “Trilogy”, designated as N3, “Boomer”, designated as N5, “Ag‐Spectrum”, designated as N6, and “Argyle”, designated as N10, *B. juncea* genotypes “JN9‐04” and “Purple leaf mustard” (J1 and J2 respectively), and *B. carinata* “195923.3.2_01DH”, and “94024.2_02DH” (C1 and C2 respectively) (Table 1). To investigate single meiotic events in these AABC and CCAB F_1_ hybrids, a combination of microspore culture (to produce plants from individual male gametes) and test crossing (to identify alleles contributed from individual female gametes in a cross‐combination with another, known paternal genotype) was previously carried out to produce this material (Figure 7). A high proportion of gametes derived from the AABC and CCAB F_1_ hybrids using both methods were found to be unreduced via a first division restitution mechanism (such as parallel spindles), and contained both sets of homologous chromosomes from the diploid genome (AA in AABC, CC in CCAB) (Figure 8, Table 1). This material was also previously described in Mason et al. (2014) where the unreduced gamete‐derived progeny were used for centromere mapping. In the present study, we further investigated homologous and non‐homologous recombination frequencies in this material (Figure 8).

**Table 1:** Genotype combinations and numbers of *Brassica* AABC and CCAB F_1_ hybrid‐derived progeny

Hybrid type	Parent 1	Parent 2	Genotype combination	No. of test cross progeny from reduced gametes	No. of test cross progeny from unreduced gametes	No. of microspore‐derived progeny from reduced gametes	No. of microspore‐derived progeny from unreduced gametes
AABC (*B. juncea* × *B. napus*)	“JN9‐04”	“Surpass400_024DH”	J1N1	14	19	—	
	“Surpass400_024DH”	“JN9‐04”	N1J1	20	—	—	—
	“JN9‐04”	“Trilogy”	J1N3	—	—	4	18
	“JN9‐04”	“Boomer”	J1N5	—	—	—	22
	“JN9‐04”	“Argyle”	J1N10	—	—	1	17
	“Purple Leaf Mustard”	“Surpass400_024DH”	J2N1	—	—	3	19
CCAB (*B. napus* × *B. carinata*)	“Trilogy”	“94024.2_02DH”	N3C2			6	30
	“Boomer”	“94024.2_02DH”	N5C2	10	14	15	5
	“Ag‐Spectrum”	“94024.2_02DH”	N6C2	4	7	4	14
	“Lynx_037DH”	“195923.3.2_01DH”	N7C1	2	3	2	19

### Genotyping using the Illumina Infinium *Brassica* 60 K SNP array

All SNP probe sequences were subject to BLAST analysis against the *B. napus* Darmor‐bzh v10 reference genome for genome position information (Boideau et al., 2022; Rousseau‐Gueutin et al., 2021). Subsequently, SNP probe sequences were screened with the following criteria: (1) filter out the sequences with lengths of less than 50 bp; (2) only include the cases where the sequence identification values were 100%; (3) only sequences with a unique match in the genome. After filtering, 35 328 SNPs were kept for further analysis (Table S1). The AABC and CCAB hybrid populations were genotyped using the Illumina Infinium *Brassica* 60 K array (Illumina Inc., USA) following the manufacturer's protocol. The genotyping data were scanned and exported by the Genome Studio software (Illumina Inc., USA). Genotype data (AA, AB, BB, and NC) along with log R ratio (logR) values were exported and compiled (Tables S2 and S3).

### Detection of chromosome variation in the AABC and CCAB unreduced gamete‐derived population

The Ascat R package was used to analyze log R ratio (logR) and B allele frequency (BAF) files (Tables S2 and S3) (Ross et al., 2021; Van Loo et al., 2010). Ascat R was used to perform segmentation and smoothing of BAF and logR values and to correct for allele‐specific copy number variation using the default built‐in linear model‐based algorithm. Plots for every individual line based on estimated cut‐off values were produced to score copy number variants. All the A‐ and C‐genome SNP information for the parent lines is shown in Figure S1.

### Detection of crossover frequency in the AABC and CCAB populations

Using these data (Table S2), we began with a standard initial filtering process, as follows: SNPs with over 70% heterozygous (AB) calls in all parent genotypes and those with more than 90% missing data across all lines were also removed. We selected only SNPs that were homozygous in and polymorphic between the individual parent genotypes of each hybrid combination in the diploid genomes (C in CCAB and A in AABC hybrids). Additionally, we retained only loci for these diploid genomes that were heterozygous in the F_1_ hybrid parent. After these steps, we converted the progeny genotypes: alleles from the *B. napus* parent were labeled as “a” and alleles from *the B. juncea* and *B. carinata* parents as “b,” heterozygous alleles were labeled as “h,” and NA calls indicating missing values remained unchanged (Figure S2). This process resulted in the final Table S4 dataset.

All the following analyses were done using basic functions of the R statistical environment (R v. 4.4.0) (R Core Team 2024). To count crossovers, the entire SNP matrix containing raw data (Table S4) was first converted into character format, and the function rle() in base R was applied to smooth the character vectors. Specifically, we replaced segments of consecutive identical values that were shorter than the defined minimum length, removing small outliers to make the data smoother and reduce fluctuations. Minimum breakpoint lengths were iteratively checked per chromosome between 3 and 30 to determine appropriate cut‐offs based on data quality: recovery of unreasonable individual breakpoint counts of >20 breakpoints per chromosome was taken to indicate data quality issues, and finally minimum lengths between 5 and 15 (5, 10, 13, and 15) were used to ensure that the final number of breakpoints was close to the true value. Additionally, if consecutive NA values exceed the defined minimum threshold, we considered them as chromosomal deletions. Then, the custom R script identifies groupings of regions that are too short to comprise crossovers with high certainty (based on a minimum distance between crossovers set by 2 Mb). After that the script follows the rules below to find crossovers and merge away regions that are too short. The script then outputs the crossover numbers per chromosome (https://github.com/zhenling0628/Brassica‐AABC‐and‐CCAB‐crossover‐number).

Non‐homologous crossovers were manually detected in 2n‐gamete‐derived individuals using changes in LogRRatio indicating the presence of AAB or BBA allele ratios in the diploid genome or by deletions (NC) indicating the loss of both parental alleles. Non‐homologous crossovers were manually detected in n‐gamete‐derived individuals using the presence of AB or NC calls or by copy number variation. As we identified a significant number of non‐homologous recombination events, we also needed to exclude these from our analysis in order to estimate the number of homologous crossovers in the AABC and CCAB populations.

### Statistical analysis

Pearson's *χ* ^2^‐test was used to test counts of chromosome variation and a one‐way ANOVA test was used to test for significant differences between different genotypes in base R v. 4.4.0.

## AUTHOR CONTRIBUTIONS

ZL and ASM analyzed and interpreted data and drafted the manuscript. SM assisted in the visualization and finalization of figures. ASM conceptualized and designed the project, acquired funding and supervised ZL and SM. ZL, SM, and ASM critically revised the manuscript.

## CONFLICT OF INTEREST

The authors declare that there are no competing interests.

## Supporting information


**Figure S1.** Example of the chromosome copy number variation pipeline in *Brassica napus, Brassica carinata*, and *Brassica juncea* parent lines.
**Figure S2.** Introduction to the genotype data workflow: genotype calling, quality control, and filtering to ensure accuracy and reliability.
**Figure S3.** Illustration of copy number variation based on haplotypes derived from the Ascat R package in the *Brassica* CCAB unreduced gamete‐derived population. (a and b) Euploid example from H1_19 (N5C2) testcross material. (c and d) Whole chromosome deletion and partial chromosome deletion examples from H1_4 (N5C2) testcross material; green arrows represent chromosome deletions. (e and f) Examples of chromosome segment deletion and gain from the N7C1 genotype 2n‐derived material. (a, c, and e) logR data. (b, d, and f) B allele frequency data; green arrows represent chromosome loss and pink arrows represent chromosome gain.
**Figure S4.** Illustration of haplotypes in the *Brassica* AABC and CCAB unreduced gamete‐derived populations. (a) AABC and (b) CCAB 2n population; red asterisks indicate individuals with the highest number of crossover events.
**Figure S5.** Spatial distribution of crossover (CO) events along the AA or CC genomes in *Brassica* AABC and CCAB hybrid‐derived populations. The *y*‐axis represents the physical position along each chromosome, while the *x*‐axis shows the number of CO events detected within 1 Mb windows. Dark yellow indicates the centromeric region, light yellow marks the pericentromeric region, and gray represents the overall chromosomal framework.
**Figure S6.** Genome‐wide crossover frequencies in *Brassica* AABC and CCAB populations. Red represents the unreduced gamete‐derived population (2n) and blue indicates the reduced gamete‐derived population (n). The vertical dashed lines indicated the average crossover value.
**Figure S7.** Correlation between chromosome length and the crossover frequency. Non‐significant correlation, *P*‐value = 0.57.

**Table S1.** The 35 328 SNPs used for analysis after filtering probe sequences for unique BLAST hits to *Brassica napus* Darmor‐bzh v10.
**Table S2.** Genotype calls for the SNPs used in the analysis of the *Brassica* AABC and CCAB hybrids.
**Table S3.** Log R ratios (logR) for the SNPs used in the analysis of the *Brassica* AABC and CCAB hybrids.
**Table S4.** SNP genotypes after converting to character vectors (a, b, and h).
**Table S5.** Chromosome‐specific copy number variant information for the *Brassica* AABC unreduced gamete‐derived population.
**Table S6.** Chromosome‐specific copy number variant information for the *Brassica* CCAB unreduced gamete‐derived population.
**Table S7.** Chromosome‐specific copy number variant information for the *Brassica* AABC reduced gamete‐derived population.
**Table S8.** Chromosome‐specific copy number variant information for the *Brassica* CCAB reduced gamete‐derived population.
**Table S9.** Crossover numbers per homologous chromosome in the A genome of the *Brassica* AABC hybrid‐derived population.
**Table S10.** Crossover numbers per homologous chromosome in the C genome of the *Brassica* CCAB hybrid‐derived population.

## Data Availability

The data that supports the findings of this study are available in the supporting information of this article or are previously published.

## References

[tpj70555-bib-0001] Adonina, I.G. , Timonova, E.M. & Salina, E.A. (2021) Introgressive hybridization of common wheat: results and prospects. Russian Journal of Genetics, 57, 390–407.

[tpj70555-bib-0002] Blary, A. , Gonzalo, A. , Eber, F. , Bérard, A. , Bergès, H. , Bessoltane, N. et al. (2018) FANCM limits meiotic crossovers in *Brassica* crops. Frontiers in Plant Science, 9, 368. 29628933 10.3389/fpls.2018.00368 PMC5876677

[tpj70555-bib-0003] Boideau, F. , Huteau, V. , Maillet, L. , Brunet, A. , Coriton, O. , Deniot, G. et al. (2024) Alternating between even and odd ploidy levels switches on and off the recombination control, even near the centromeres. The Plant Cell, 36, 4472–4490. 39121028 10.1093/plcell/koae208 PMC11449113

[tpj70555-bib-0004] Boideau, F. , Pelé, A. , Tanguy, C. , Trotoux, G. , Eber, F. , Maillet, L. et al. (2021) A modified meiotic recombination in *Brassica napus* largely improves its breeding efficiency. Biology, 10, 771. 34440003 10.3390/biology10080771 PMC8389541

[tpj70555-bib-0005] Boideau, F. , Richard, G. , Coriton, O. , Huteau, V. , Belser, C. , Deniot, G. et al. (2022) Epigenomic and structural events preclude recombination in *Brassica napus* . New Phytologist, 234, 545–559. 35092024 10.1111/nph.18004

[tpj70555-bib-0006] Cai, C. , Pelé, A. , Bucher, J. , Finkers, R. & Bonnema, G. (2023) Fine mapping of meiotic crossovers in *Brassica oleracea* reveals patterns and variations depending on direction and combination of crosses. The Plant Journal, 113, 1192–1210. 36626115 10.1111/tpj.16104

[tpj70555-bib-0007] Chalhoub, B. , Denoeud, F. , Liu, S. , Parkin, I.A.P. , Tang, H. , Wang, X. et al. (2014) Early allopolyploid evolution in the post‐neolithic *Brassica napus* oilseed genome. Science, 345, 950–953. 25146293 10.1126/science.1253435

[tpj70555-bib-0008] Cheng, F. , Mandáková, T. , Wu, J. , Xie, Q. , Lysak, M.A. & Wang, X. (2013) Deciphering the diploid ancestral genome of the Mesohexaploid *Brassica rapa* . The Plant Cell, 25, 1541–1554. 23653472 10.1105/tpc.113.110486 PMC3694691

[tpj70555-bib-0009] Choi, K. & Henderson, I.R. (2015) Meiotic recombination hotspots—a comparative view. The Plant Journal, 83, 52–61. 25925869 10.1111/tpj.12870

[tpj70555-bib-0010] Cowling, W.A. (2007) Genetic diversity in Australian canola and implications for crop breeding for changing future environments. Field Crops Research, 104, 103–111.

[tpj70555-bib-0011] Dreissig, S. , Maurer, A. , Sharma, R. , Milne, L. , Flavell, A.J. , Schmutzer, T. et al. (2020) Natural variation in meiotic recombination rate shapes introgression patterns in intraspecific hybrids between wild and domesticated barley. New Phytologist, 228, 1852–1863. 32659029 10.1111/nph.16810

[tpj70555-bib-0012] Gaebelein, R. , Alnajar, D. , Koopmann, B. & Mason, A.S. (2019) Hybrids between *Brassica napus* and *B. nigra* show frequent pairing between the B and a/C genomes and resistance to blackleg. Chromosome Research, 27, 221–236. 31280459 10.1007/s10577-019-09612-2

[tpj70555-bib-0013] Gaebelein, R. & Mason, A.S. (2018) Allohexaploids in the genus *Brassica* . Critical Reviews in Plant Sciences, 37, 422–437.

[tpj70555-bib-0014] Gaebelein, R. , Schiessl, S.V. , Samans, B. , Batley, J. & Mason, A.S. (2019) Inherited allelic variants and novel karyotype changes influence fertility and genome stability in *Brassica* allohexaploids. New Phytologist, 223, 965–978. 30887525 10.1111/nph.15804

[tpj70555-bib-0015] Gaeta, R.T. & Chris Pires, J. (2010) Homoeologous recombination in allopolyploids: the polyploid ratchet. New Phytologist, 186, 18–28. 20002315 10.1111/j.1469-8137.2009.03089.x

[tpj70555-bib-0016] Gaeta, R.T. , Pires, J.C. , Iniguez‐Luy, F. , Leon, E. & Osborn, T.C. (2007) Genomic changes in resynthesized *Brassica napus* and their effect on gene expression and phenotype. The Plant Cell, 19, 3403–3417. 18024568 10.1105/tpc.107.054346 PMC2174891

[tpj70555-bib-0017] Higgins, E.E. , Howell, E.C. , Armstrong, S.J. & Parkin, I.A.P. (2021) A major quantitative trait locus on chromosome A9, BnaPh1, controls homoeologous recombination in *Brassica napus* . New Phytologist, 229, 3281–3293. 33020949 10.1111/nph.16986 PMC7984352

[tpj70555-bib-0018] Hu, J. , Chen, B. , Zhao, J. , Zhang, F. , Xie, T. , Xu, K. et al. (2022) Genomic selection and genetic architecture of agronomic traits during modern rapeseed breeding. Nature Genetics, 54, 694–704. 35484301 10.1038/s41588-022-01055-6

[tpj70555-bib-0019] Ihien Katche, E. & S. Mason, A. (2023) Resynthesized rapeseed (*Brassica napus*): breeding and genomics. Critical Reviews in Plant Sciences, 42, 65–92.

[tpj70555-bib-0020] Jenczewski, E. , Eber, F. , Grimaud, A. , Huet, S. , Lucas, M.O. , Monod, H. et al. (2003) PrBn, a major gene controlling Homeologous pairing in oilseed rape (*Brassica napus*) haploids. Genetics, 164, 645–653. 12807785 10.1093/genetics/164.2.645 PMC1462591

[tpj70555-bib-0021] Katche, E.I. , Schierholt, A. , Becker, H.C. , Batley, J. & Mason, A.S. (2023) Fertility, genome stability, and homozygosity in a diverse set of resynthesized rapeseed lines. The Crop Journal, 11, 468–477.

[tpj70555-bib-0022] Katche, E.I. , Schierholt, A. , Schiessl, S.‐V. , He, F. , Lv, Z. , Batley, J. et al. (2023) Genetic factors inherited from both diploid parents interact to affect genome stability and fertility in resynthesized allotetraploid *Brassica napus* . G3: Genes, Genomes, Genetics, 13, jkad136. 37313757 10.1093/g3journal/jkad136 PMC10411605

[tpj70555-bib-0023] Katche, E. , Quezada‐Martinez, D. , Katche, E.I. , Vasquez‐Teuber, P. & Mason, A.S. (2019) Interspecific hybridization for *Brassica* crop improvement. Crop Breeding, Genetics and Genomics, 1, e190007.

[tpj70555-bib-0024] Kreiner, J.M. , Kron, P. & Husband, B.C. (2017) Evolutionary dynamics of unreduced gametes. Trends in Genetics, 33, 583–593. 28732599 10.1016/j.tig.2017.06.009

[tpj70555-bib-0025] Leflon, M. , Grandont, L. , Eber, F. , Huteau, V. , Coriton, O. , Chelysheva, L. et al. (2010) Crossovers get a boost in *Brassica* allotriploid and allotetraploid hybrids. The Plant Cell, 22, 2253–2264. 20622148 10.1105/tpc.110.075986 PMC2929096

[tpj70555-bib-0026] Liu, S. , Liu, Y. , Yang, X. , Tong, C. , Edwards, D. , Parkin, I.A.P.P. et al. (2014) The *Brassica oleracea* genome reveals the asymmetrical evolution of polyploid genomes. Nature Communications, 5, 3930. 10.1038/ncomms4930 PMC4279128 24852848

[tpj70555-bib-0027] Liu, Z. , Adamczyk, K. , Manzanares‐Dauleux, M. , Eber, F. , Lucas, M.‐O. , Delourme, R. et al. (2006) Mapping *PrBn* and other quantitative trait loci responsible for the control of homeologous chromosome pairing in oilseed rape (*Brassica napus* L.) haploids. Genetics, 174, 1583–1596. 16951054 10.1534/genetics.106.064071 PMC1667100

[tpj70555-bib-0028] Lysak, M.A. , Koch, M.A. , Pecinka, A. & Schubert, I. (2005) Chromosome triplication found across the tribe *Brassiceae* . Genome Research, 15, 516–525. 15781573 10.1101/gr.3531105 PMC1074366

[tpj70555-bib-0029] Mason, A.S. , Batley, J. , Bayer, P.E. , Hayward, A. , Cowling, W.A. & Nelson, M.N. (2014) High‐resolution molecular karyotyping uncovers pairing between ancestrally related *Brassica* chromosomes. New Phytologist, 202, 964–974. 24471809 10.1111/nph.12706

[tpj70555-bib-0030] Mason, A.S. , Huteau, V. , Eber, F. , Coriton, O. , Yan, G. , Nelson, M.N. et al. (2010) Genome structure affects the rate of autosyndesis and allosyndesis in AABC, BBAC and CCAB *Brassica* interspecific hybrids. Chromosome Research, 18, 655–666. 20571876 10.1007/s10577-010-9140-0

[tpj70555-bib-0031] Mason, A.S. , Nelson, M.N. , Castello, M.C. , Yan, G. & Cowling, W.A. (2011) Genotypic effects on the frequency of homoeologous and homologous recombination in *Brassica napus × B. carinata* hybrids. Theoretical and Applied Genetics, 122, 543–553. 21046065 10.1007/s00122-010-1468-5

[tpj70555-bib-0032] Mason, A.S. , Nelson, M.N. , Yan, G. & Cowling, W.A. (2011) Production of viable male unreduced gametes in *Brassica* interspecific hybrids is genotype specific and stimulated by cold temperatures. BMC Plant Biology, 11, 103. 21663695 10.1186/1471-2229-11-103 PMC3141635

[tpj70555-bib-0033] Mason, A.S. & Pires, J.C. (2015) Unreduced gametes: meiotic mishap or evolutionary mechanism? Trends in Genetics, 31, 5–10. 25445549 10.1016/j.tig.2014.09.011

[tpj70555-bib-0034] Mason, A.S. , Rousseau‐Gueutin, M. , Morice, J. , Bayer, P.E. , Besharat, N. , Cousin, A. et al. (2016) Centromere locations in *Brassica* a and C genomes revealed through half‐tetrad analysis. Genetics, 202, 513–523. 26614742 10.1534/genetics.115.183210 PMC4788232

[tpj70555-bib-0035] Mason, A.S. & Snowdon, R.J. (2016) Oilseed rape: learning about ancient and recent polyploid evolution from a recent crop species. Plant Biology, 18, 883–892. 27063780 10.1111/plb.12462

[tpj70555-bib-0036] Mason, A.S. , Takahira, J. , Atri, C. , Samans, B. , Hayward, A. , Cowling, W.A. et al. (2015) Microspore culture reveals complex meiotic behaviour in a trigenomic *Brassica* hybrid. BMC Plant Biology, 15, 1–15. 26152188 10.1186/s12870-015-0555-9 PMC4493989

[tpj70555-bib-0037] Mercier, R. , Mézard, C. , Jenczewski, E. , Macaisne, N. & Grelon, M. (2015) The molecular biology of meiosis in plants. Annual Review of Plant Biology, 66, 297–327. 10.1146/annurev-arplant-050213-035923 25494464

[tpj70555-bib-0038] Mézard, C. , Tagliaro Jahns, M. & Grelon, M. (2015) Where to cross? New insights into the location of meiotic crossovers. Trends in Genetics, 31, 393–401. 25907025 10.1016/j.tig.2015.03.008

[tpj70555-bib-0039] Mwathi, M.W. , Gupta, M. , Atri, C. , Banga, S.S. , Batley, J. & Mason, A.S. (2017) Segregation for fertility and meiotic stability in novel *Brassica* allohexaploids. Theoretical and Applied Genetics, 130, 767–776. 28097399 10.1007/s00122-016-2850-8

[tpj70555-bib-0040] Nelson, M.N. , Mason, A.S. , Castello, M.C. , Thomson, L. , Yan, G. & Cowling, W.A. (2009) Microspore culture preferentially selects unreduced (2n) gametes from an interspecific hybrid of *Brassica napus L. × Brassica carinata Braun* . Theoretical and Applied Genetics, 119, 497–505. 19436985 10.1007/s00122-009-1056-8

[tpj70555-bib-0041] Nicolas, S.D. , Leflon, M. , Monod, H. , Eber, F. , Coriton, O. , Huteau, V. et al. (2009) Genetic regulation of meiotic cross‐overs between related genomes in *Brassica napus* haploids and hybrids. The Plant Cell, 21, 373–385. 19190241 10.1105/tpc.108.062273 PMC2660629

[tpj70555-bib-0042] Osborn, T.C. , Butrulle, D.V. , Sharpe, A.G. , Pickering, K.J. , Parkin, I.A.P. , Parker, J.S. et al. (2003) Detection and effects of a Homeologous reciprocal transposition in *Brassica napus* . Genetics, 165, 1569–1577. 14668403 10.1093/genetics/165.3.1569 PMC1462855

[tpj70555-bib-0043] Parkin, I.A.P. , Sharpe, A.G. , Keith, D.J. & Lydiate, D.J. (1995) Identification of the a and C genomes of amphidiploid *Brassica napus* (oilseed rape). Genome, 38, 1122–1131. 18470236 10.1139/g95-149

[tpj70555-bib-0044] Parkin, I.A.P. , Sharpe, A.G. & Lydiate, D.J. (2003) Patterns of genome duplication within the *Brassica napus* genome. Genome, 46, 291–303. 12723045 10.1139/g03-006

[tpj70555-bib-0045] Pelé, A. , Falque, M. , Lodé‐Taburel, M. , Huteau, V. , Morice, J. , Coriton, O. et al. (2025) Genomic divergence shaped the genetic regulation of meiotic homologous recombination in *Brassica* allopolyploids. Molecular Biology and Evolution, 42, msaf073. 40173423 10.1093/molbev/msaf073 PMC11982612

[tpj70555-bib-0046] Pelé, A. , Falque, M. , Trotoux, G. , Eber, F. , Nègre, S. , Gilet, M. et al. (2017) Amplifying recombination genome‐wide and reshaping crossover landscapes in *Brassicas* . PLoS Genetics, 13, e1006794. 28493942 10.1371/journal.pgen.1006794 PMC5444851

[tpj70555-bib-0047] Pelé, A. , Rousseau‐Gueutin, M. & Chèvre, A.‐M. (2018) Speciation success of polyploid plants closely relates to the regulation of meiotic recombination. Frontiers in Plant Science, 9, 1–9. 30002669 10.3389/fpls.2018.00907 PMC6031745

[tpj70555-bib-0048] Quezada‐Martinez, D. , Addo Nyarko, C.P. , Schiessl, S.V. & Mason, A.S. (2021) Using wild relatives and related species to build climate resilience in *Brassica* crops. Theoretical and Applied Genetics, 134, 1711–1728. 33730183 10.1007/s00122-021-03793-3 PMC8205867

[tpj70555-bib-0049] Quezada‐Martinez, D. , Zou, J. , Zhang, W. , Meng, J. , Batley, J. & Mason, A.S. (2022) Allele segregation analysis of F1 hybrids between independent *Brassica* allohexaploid lineages. Chromosoma, 131, 147–161. 35511360 10.1007/s00412-022-00774-3 PMC9470611

[tpj70555-bib-0150] R Core Team (2024) *R: a language and environment for statistical computing.* Vienna, Austria: R Foundation for Statistical Computing. https://www.R-project.org/.

[tpj70555-bib-0050] Ramsey, J. & Schemske, D.W. (1998) Pathways, mechanisms, and rates of polyploid formation in flowering plants. Annual Review of Ecology and Systematics, 29, 467–501.

[tpj70555-bib-0051] Ross, E.M. , Haase, K. , Van Loo, P. & Markowetz, F. (2021) Allele‐specific multi‐sample copy number segmentation in ASCAT. Bioinformatics, 37, 1909–1911. 32449758 10.1093/bioinformatics/btaa538 PMC8317109

[tpj70555-bib-0052] Rousseau‐Gueutin, M. , Belser, C. , Da, S.C. , Richard, G. , Istace, B. , Cruaud, C. et al. (2021) Long‐read assembly of the *Brassica napus* reference genome *Darmor‐bzh* . GigaScience, 9, 1–16. 10.1093/gigascience/giaa137 PMC7736779 33319912

[tpj70555-bib-0053] Rousseau‐Gueutin, M. , Morice, J. , Coriton, O. , Huteau, V. , Trotoux, G. , Nègre, S. et al. (2017) The impact of open pollination on the structural evolutionary dynamics, meiotic behavior, and fertility of resynthesized allotetraploid *Brassica napus L* . G3: Genes, Genomes, Genetics, 7, 705–717. 28007837 10.1534/g3.116.036517 PMC5295613

[tpj70555-bib-0054] Schiessl, S.V. , Katche, E. , Ihien, E. , Chawla, H.S. & Mason, A.S. (2019) The role of genomic structural variation in the genetic improvement of polyploid crops. The Crop Journal, 7, 127–140.

[tpj70555-bib-0055] Schranz, M.E. , Lysak, M.A. & Mitchell‐Olds, T. (2006) The ABC's of comparative genomics in the *Brassicaceae*: building blocks of crucifer genomes. Trends in Plant Science, 11, 535–542. 17029932 10.1016/j.tplants.2006.09.002

[tpj70555-bib-0056] Schranz, M.E. & Osborn, T.C. (2000) Novel flowering time variation in the resynthesized polyploid *Brassica napus* . Journal of Heredity, 91, 242–246. 10833052 10.1093/jhered/91.3.242

[tpj70555-bib-0057] Song, K. , Lu, P. , Tang, K. & Osborn, T.C. (1995) Rapid genome change in synthetic polyploids of *Brassica* and its implications for polyploid evolution. Proceedings of the National Academy of Sciences, 92, 7719–7723. 10.1073/pnas.92.17.7719 PMC41217 7644483

[tpj70555-bib-0058] Stein, A. , Coriton, O. , Rousseau‐Gueutin, M. , Samans, B. , Schiessl, S.V. , Obermeier, C. et al. (2017) Mapping of homoeologous chromosome exchanges influencing quantitative trait variation in *Brassica napus* . Plant Biotechnology Journal, 15, 1478–1489. 28370938 10.1111/pbi.12732 PMC5633767

[tpj70555-bib-0059] Suay, L. , Zhang, D. , Eber, F. , Jouy, H. , Lodé, M. , Huteau, V. et al. (2014) Crossover rate between homologous chromosomes and interference are regulated by the addition of specific unpaired chromosomes in *Brassica* . New Phytologist, 201, 645–656. 24117470 10.1111/nph.12534

[tpj70555-bib-0060] Szadkowski, E. , Eber, F. , Huteau, V. , Lodé, M. , Coriton, O. , Jenczewski, E. et al. (2011) Polyploid formation pathways have an impact on genetic rearrangements in resynthesized *Brassica napus* . New Phytologist, 191, 884–894. 21517871 10.1111/j.1469-8137.2011.03729.x

[tpj70555-bib-0061] Szadkowski, E. , Eber, F. , Huteau, V. , Lodé, M. , Huneau, C. , Belcram, H. et al. (2010) The first meiosis of resynthesized *Brassica napus*, a genome blender. New Phytologist, 186, 102–112. 20149113 10.1111/j.1469-8137.2010.03182.x

[tpj70555-bib-0062] Takahira, J. , Cousin, A. , Nelson, M.N. & Cowling, W.A. (2011) Improvement in efficiency of microspore culture to produce doubled haploid canola (*Brassica napus L*.) by flow cytometry. Plant Cell, Tissue and Organ Culture, 104, 51–59.

[tpj70555-bib-0063] Van Loo, P. , Nordgard, S.H. , Lingjærde, O.C. , Russnes, H.G. , Rye, I.H. , Sun, W. et al. (2010) Allele‐specific copy number analysis of tumors. Proceedings of the National Academy of Sciences, 107, 16910–16915. 10.1073/pnas.1009843107 PMC2947907 20837533

[tpj70555-bib-0064] Wang, B. , Wu, Z. , Li, Z. , Zhang, Q. , Hu, J. , Xiao, Y. et al. (2018) Dissection of the genetic architecture of three seed‐quality traits and consequences for breeding in *Brassica napus* . Plant Biotechnology Journal, 16, 1336–1348. 29265559 10.1111/pbi.12873 PMC5999192

[tpj70555-bib-0065] Wang, T. , Van Dijk, A.D.J. , Bucher, J. , Liang, J. , Wu, J. , Bonnema, G. et al. (2023) Interploidy introgression shaped adaptation during the origin and domestication history of *Brassica napus* . Molecular Biology and Evolution, 40, 1–16. 10.1093/molbev/msad199 PMC10504873 37707440

[tpj70555-bib-0066] Wang, X. , Wang, H. , Wang, J. , Sun, R. , Wu, J. , Liu, S. et al. (2011) The genome of the mesopolyploid crop species *Brassica rapa* . Nature Genetics, 43, 1035–1040. 21873998 10.1038/ng.919

[tpj70555-bib-0067] Xiong, Z. , Gaeta, R.T. & Pires, J.C. (2011) Homoeologous shuffling and chromosome compensation maintain genome balance in resynthesized allopolyploid *Brassica napus* . Proceedings of the National Academy of Sciences, 108, 7908–7913. 10.1073/pnas.1014138108 PMC3093481 21512129

[tpj70555-bib-0068] Yang, F. , Wan, H. , Li, J. , Wang, Q. , Yang, N. , Zhu, X. et al. (2022) Pentaploidization enriches the genetic diversity of wheat by enhancing the recombination of AB genomes. Frontiers in Plant Science, 13, 883868. 35845672 10.3389/fpls.2022.883868 PMC9281561

[tpj70555-bib-0069] Yu, J. , Tehrim, S. , Wang, L. , Dossa, K. , Zhang, X. , Ke, T. et al. (2017) Evolutionary history and functional divergence of the cytochrome P450 gene superfamily between *Arabidopsis thaliana* and *Brassica* species uncover effects of whole genome and tandem duplications. BMC Genomics, 18, 1–21. 28923019 10.1186/s12864-017-4094-7 PMC5604286

