# Supplementary material for: Substantial non‐homologous recombination and structural variation results from Brassica AABC and CCAB hybrid meiosis
Source: Plant J. 2025 Oct 31;124(3):e70555. doi: 10.1111/tpj.70555 (PMC12578466; doi:10.1111/tpj.70555)
Supplement: Supplementary file 1 — Figure S1. Example of the chromosome copy number variation pipeline in Brassica napus, Brassica carinata, and Brassica juncea parent lines. Figure S2. Introduction to the genotype data workflow: genotype calling, quality control, and filtering to ensure accuracy and reliability. Figure S3. Illustration of copy number variation based on haplotypes derived from the Ascat R package in the Brassica CCAB unreduced gamete‐derived population. (a and b) Euploid example from H1_19 (N5C2) testcross material. (c and d) Whole chromosome deletion and partial chromosome deletion examples from H1_4 (N5C2) testcross material; green arrows represent chromosome deletions. (e and f) Examples of chromosome segment deletion and gain from the N7C1 genotype 2n‐derived material. (a, c, and e) logR data. (b, d, and f) B allele frequency data; green arrows represent chromosome loss and pink arrows represent chromosome gain. Figure S4. Illustration of haplotypes in the Brassica AABC and CCAB unreduced gamete‐derived populations. (a) AABC and (b) CCAB 2n population; red asterisks indicate individuals with the highest number of crossover events. Figure S5. Spatial distribution of crossover (CO) events along the AA or CC genomes in Brassica AABC and CCAB hybrid‐derived populations. The y‐axis represents the physical position along each chromosome, while the x‐axis shows the number of CO events detected within 1 Mb windows. Dark yellow indicates the centromeric region, light yellow marks the pericentromeric region, and gray represents the overall chromosomal framework. Figure S6. Genome‐wide crossover frequencies in Brassica AABC and CCAB populations. Red represents the unreduced gamete‐derived population (2n) and blue indicates the reduced gamete‐derived population (n). The vertical dashed lines indicated the average crossover value. Figure S7. Correlation between chromosome length and the crossover frequency. Non‐significant correlation, P‐value = 0.57. [file TPJ-124-0-s001.pdf]

Substantial non-homologous recombination and structural variation results from *Brassica* AABC and CCAB hybrid meiosis

Authors: Zhenling Lv[1], Shima Mahmoudi[1], and Annaliese S. Mason[1*]

[1] Plant Breeding Department, INRES, University of Bonn, Kirschallee 1, 53115 Bonn, Germany

The following Supporting Information is available for this article:

Figure S1 Example of the chromosome copy number variation pipeline in *Brassica napus*, *Brassica carinata*, and *Brassica juncea* parent lines.

Figure S2 Introduction to the genotype data workflow: genotype calling, quality control, and filtering to ensure accuracy and reliability.

Figure S3 Illustration of copy number variation based on haplotypes derived from the Ascat R package in the *Brassica* CCAB unreduced gamete-derived population. A and B: euploid example from H1_19 (N5C2) testcross material; C and D: whole chromosome deletion and partial chromosome deletion examples from H1_4 (N5C2) testcross material; green arrows represent chromosome deletions; E and F: examples of chromosome segment deletion and gain from the N7C1 genotype 2n-derived material; A, C and E: logR data; B, D and F: B allele frequency data; green arrows represent chromosome loss and pink arrows represent chromosome gain.

Figure S4 Illustration of haplotypes in the *Brassica* AABC and CCAB unreduced gamete-derived populations. A: AABC and B: CCAB 2n population; red asterisks indicate individuals with the highest number of crossover events.

Figure S5 Spatial distribution of crossover (CO) events along the AA or CC genomes in *Brassica* AABC and CCAB-hybrid derived populations. The y-axis represents the physical position along each chromosome, while the x-axis shows the number of CO events detected

within 1 Mb windows. Dark yellow indicates the centromeric region, light yellow marks the pericentromeric region, and gray represents the overall chromosomal framework.

Figure S6 Genome-wide crossover frequencies in *Brassica* AABC and CCAB populations. Red represents the unreduced gamete-derived population (2n) and blue indicates the reduced gamete-derived population (n). The vertical dashed lines indicated the average crossover value.

Figure S7 Correlation between chromosome length and the crossover frequency. Non-significant correlation, p-value = 0.57.

AACC, BBCC and AABB genome-wide chromosome variation

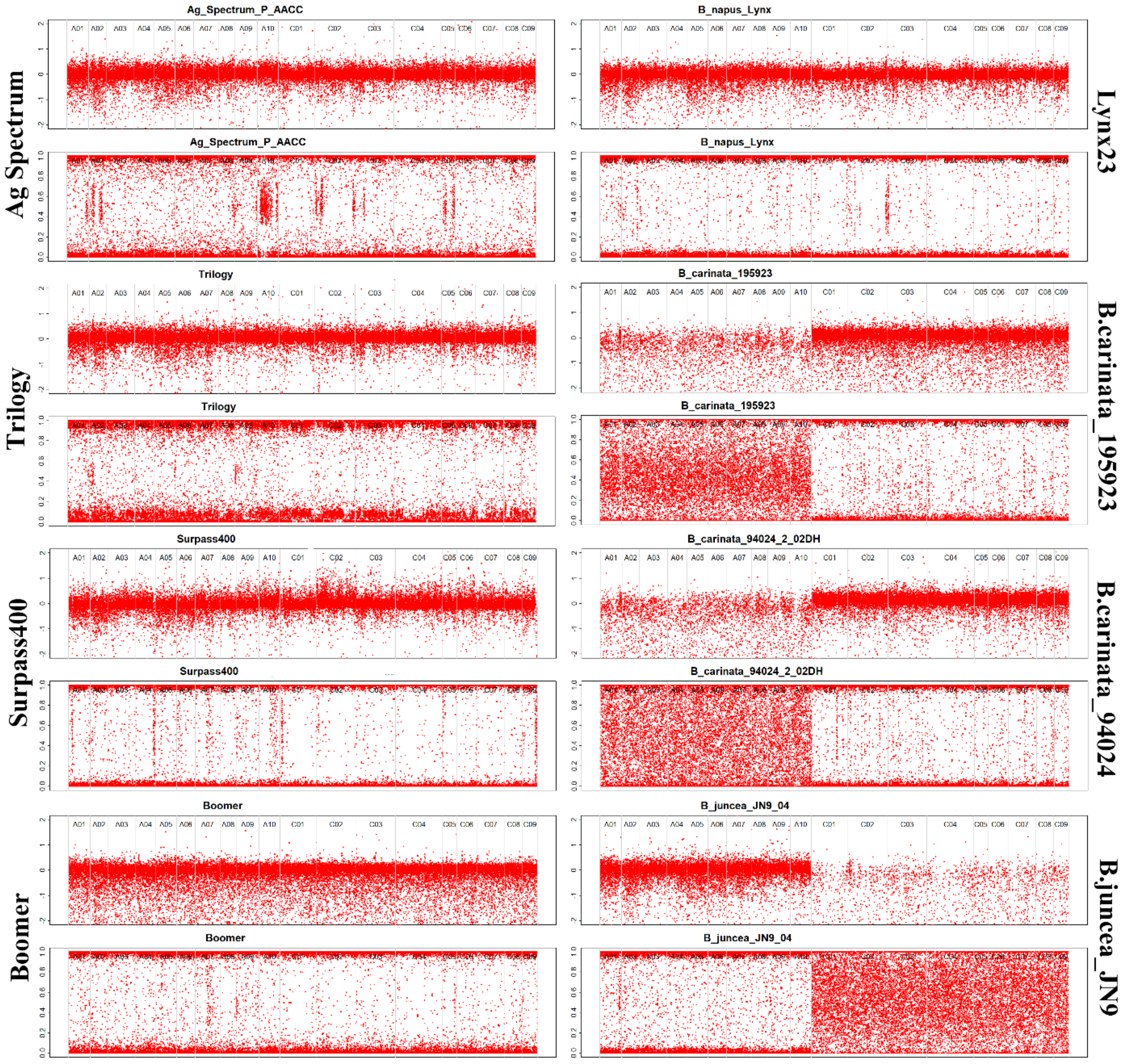

Figure S1 Example of the chromosome copy number variation pipeline in *Brassica napus*, *Brassica carinata*, and *Brassica juncea* parent lines.

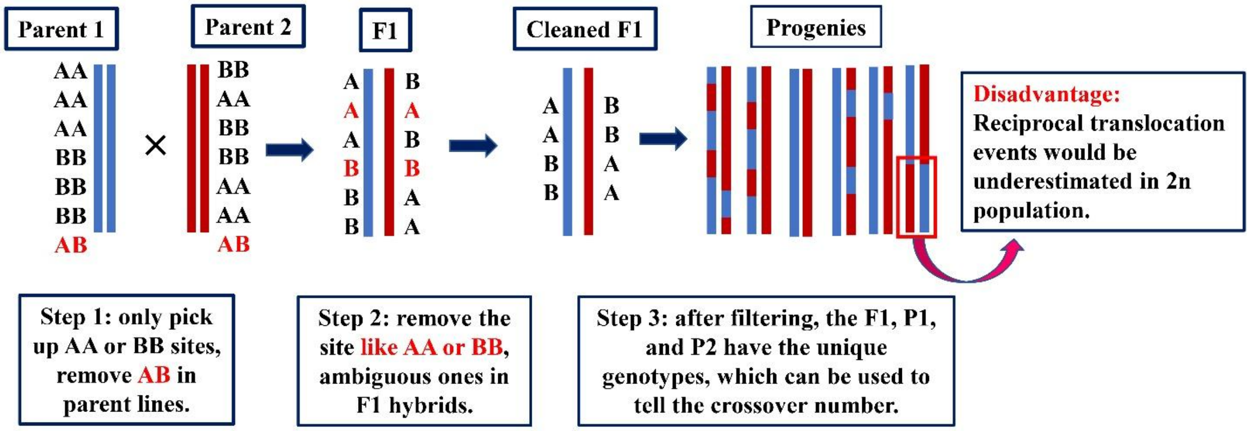

Figure S2 Introduction to the genotype data workflow: genotype calling, quality control, and filtering to ensure accuracy and reliability.

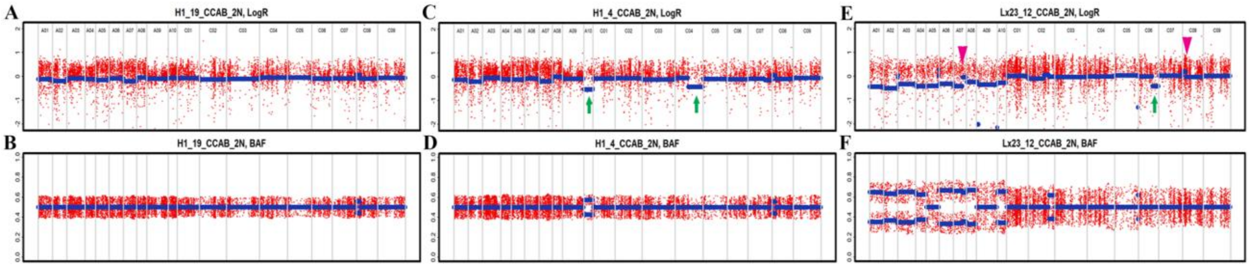

Figure S3 Illustration of copy number variation based on haplotypes derived from the Ascat R package in the *Brassica* CCAB unreduced gamete-derived population. A and B: euploid example from H1_19 (N5C2) testcross material; C and D: whole chromosome deletion and partial chromosome deletion examples from H1_4 (N5C2) testcross material; green arrows represent chromosome deletions; E and F: examples of chromosome segment deletion and gain from the N7C1 genotype 2n-derived material; A, C and E: logR data; B, D and F: B allele frequency data; green arrows represent chromosome loss and pink arrows represent chromosome gain.

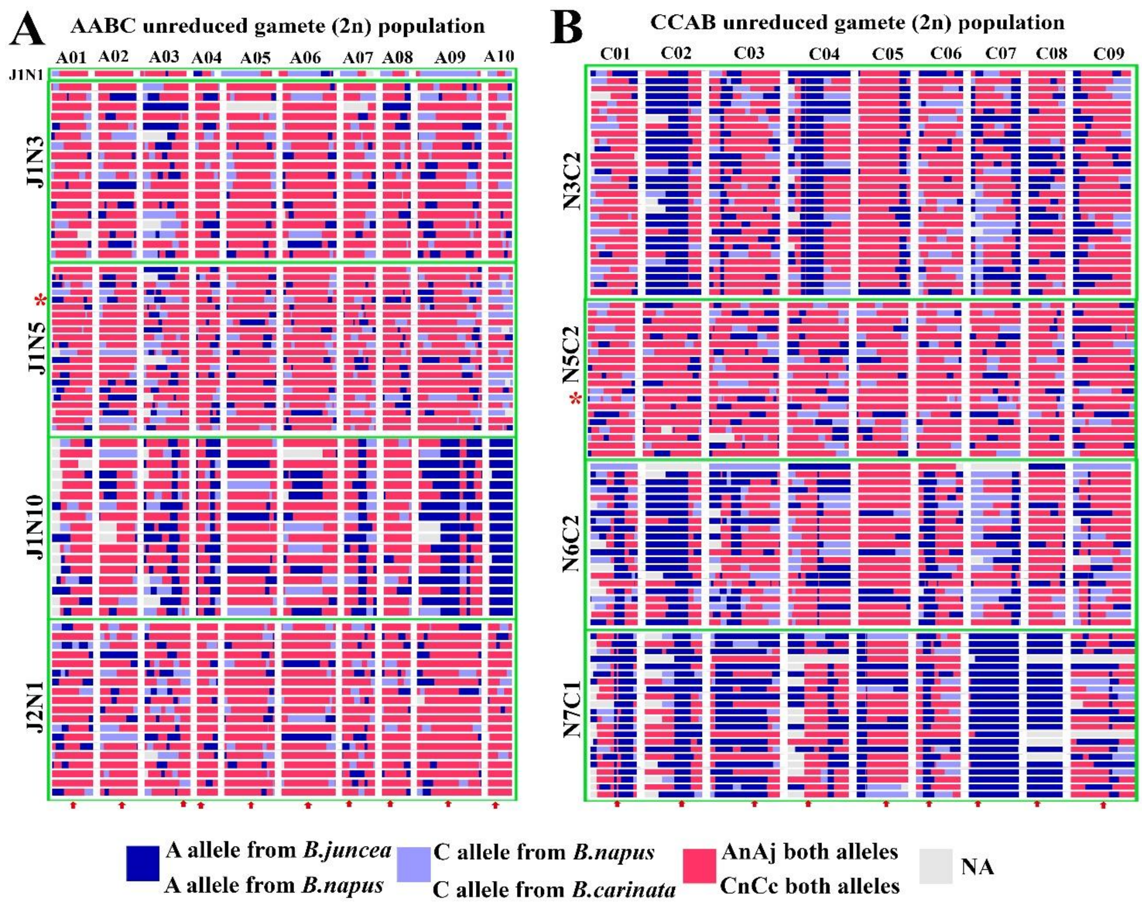

Figure S4 Illustration of haplotypes in the *Brassica* AABC and CCAB unreduced gamete-derived populations. A: AABC and B: CCAB 2n population; red asterisks indicate individuals with the highest number of crossover events.

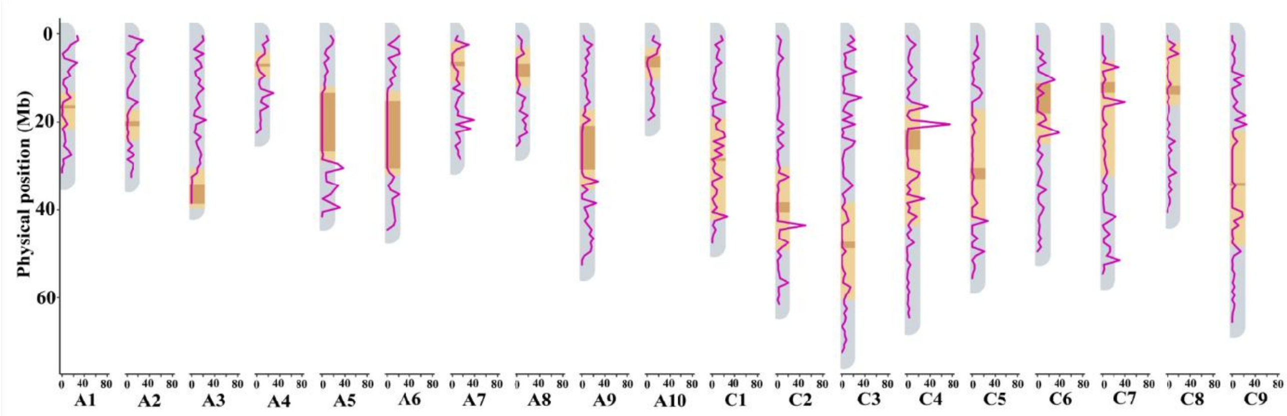

Figure S5 Spatial distribution of crossover (CO) events along the AA or CC genomes in *Brassica* AABC and CCAB-hybrid derived populations. The y-axis represents the physical position along each chromosome, while the x-axis shows the number of CO events detected within 1 Mb windows. Dark yellow indicates the centromeric region, light yellow marks the pericentromeric region, and gray represents the overall chromosomal framework.

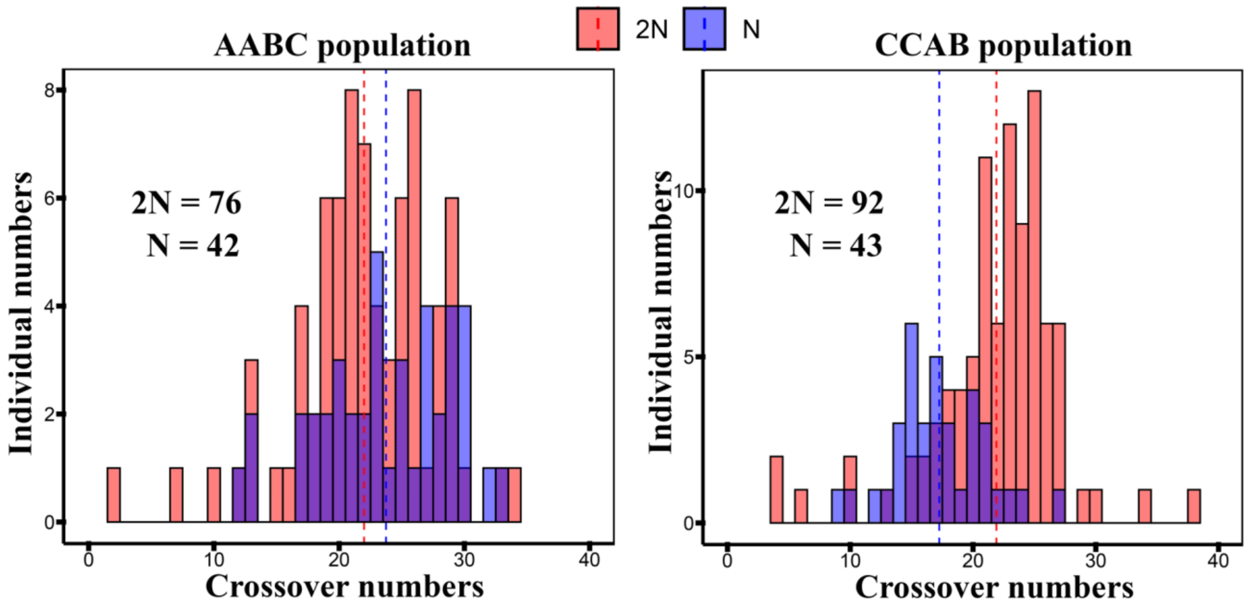

Figure S6 Genome-wide crossover frequencies in *Brassica* AABC and CCAB populations. Red represents the unreduced gamete-derived population (2n) and blue indicates the reduced gamete-derived population (n). The vertical dashed lines indicated the average crossover value.

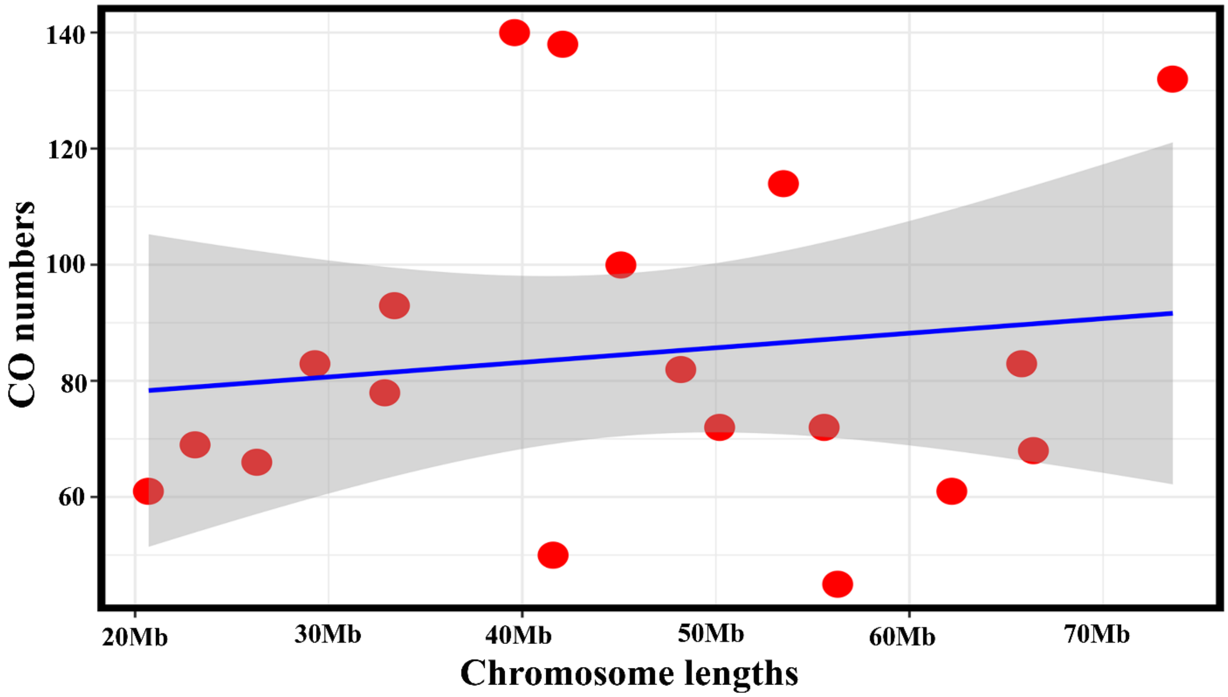

Figure S7 Correlation between chromosome length and the crossover frequency. Non-significant correlation, p-value = 0.57.
